# Supplementary material for: Relationship between socio-demographics, body composition, emotional state, and social support on metabolic syndrome risk among adults in rural Mongolia
Source: PLoS One. 2021 Sep 27;16(9):e0254141. doi: 10.1371/journal.pone.0254141 (PMC8475977; doi:10.1371/journal.pone.0254141)
Supplement: S1 Appendix — (PDF) [file pone.0254141.s001.pdf]

Монгол улс Метаболик синдром менежментийн суурь судалгаа (Mongolian Version.)

ID			
Таны хүйс	Эр ( ), Эм ( )		Таны хүйс
Биеийн хэмжилт	Өндөр	cm	Өндөр
	Биеийн жин	kg	Биеийн жин
	Хэвлийн тойрог	cm	Хэвлийн тойрог
	Даралт	mmHg	Даралт
	Пульс	beats/min	Пульс
Cholestech LDX	Цусан дахь сахар<br>(Glucose)		Цусан дахь сахар
	Холестрол<br>(Total Cholesterol)		Холестрол
	Триглецерид<br>(Triglyceride)		Триглецерид

Memo	Inbody QR

Монгол улс, Метаболик синдром менежментийн суурь судалгаа

Ерөнхий онцлог

1. Таны нар? ______нас

2. Таны хүйс? ① Эр ② Эм

3. Таны мэргэжил?
 ① Professionals (Багш, эмч, сувилагч, өмгөөлөгч гэх мэт)
 ② Technicians and associate professionals
 ③ Clerks
 ④ Service workers
 ⑤ Skilled agricultural and fishery workers
 ⑥ Мэргэжилгүй
 ⑦ Бусад (____________________________)

4. Таны гэр бүлийн сарын дундаж орлого? _________________MNT

5. Таны боловсрол?
 ① Боловсролгүй
 ② Бага боловсрол (дундаас нь орхих хамаарна)
 ③ Дунд сургууль төгссөн (Дундаас нь орхих хамаарна)
 ④ Ахлах сургууль төгссөн (Дундаас нь орхих хамаарна)
 ⑤ Баклавар (Магистер, Доктор) төгссөн(Дундаас нь орхих, хамгаалах хамаарна)

6. Гэрлэлтийн байдал? ① Нөхөр/эхнэр байгаа ② ганц бие

Өвчлөлийн судалгаа

1. Дараах өвчнөөр оношлогдож байсан эсвэл одоо эмийн эмчилгээ хийлгэж байгаа юу?

Өвчний нэр	Цус харвалт (Тархины цус харвалт)	Зүрхний өвчин (Зүрхний шигдээс)	Амьсгалын эрхтэний өвчин (Уушигны хатгаа/Астма)	Цусны даралт ихсэх	Чихрийн шижин	Элэгний өвчин (Элэгний хатуурал, элэгний үрэвсэл)
Оношлогдсон эсэх						
Эмийн эмчилгээ хийлгэж байгаа эсэх						

Бусад өвчин:_____________________________

2. Ердийн үед таны эрүүл мэнд ямар байдаг гэж боддог вэ?
 ① Маш сайн ② Сайн ③ Хэвийн ④ Муу ⑤ Маш муу

Архи согтууруулах ундаа

1. Дараах асуулт нь сүүлийн 1 жилийн согтууруулах ундаа (архи) хэрэглэж байсан талаарх асуулт юм.
   1-1. Архийг хэр ойрхон хэрэглэдэг вэ?
   ① Сүүлийн нэг жилд огт уугаагүй
   ② Сард нэг ба түүнээс бага
   ③ Сард нэг удаа
   ④ Сард 2-4 удаа
   ⑤ Долоо хоногт 2-3 удаа орчим
   ⑥ Долоо хоногт 4-өөс дээш удаа

1-2. Нэг удаадаа хэр их архи уудаг вэ?
※ Сөжү, барууны архи ялгаагүй хундагаар тооцно.
1 ширхэг лаазтай шар айраг (355cc)шар айрагны 1.6 хундагатай ижил.
① 1-2 хундага
② 3-4 хундага
③ 5-6 хундага
④ 7-9 хундага
⑤ 10-с дээш хундага

Тамхи татах

1. Одоо тамхи татдаг уу?
① Өдөр бүр татдаг → 1-1. Өдөрт дунджаар хэдэн тамхи татдаг вэ? ________ширхэг
② Хааяа татдаг
③ Өмнө нь татаж байсан боловч одоо татдаггүй.
④ Би өмнө нь үүнийг хэзээ ч хийж байгаагүй.

2. Цаашид 1 сарын хугацаанд тамхи татахаа болих төлөвлөгөө байгаа юу?
① 1 сарын дотор тамхийг хаях төлөвлөгөөтэй.
② 6 сарын дотор хаях төлөвлөгөөтэй.
③ 6 сарын дотор биш ч гэсэн хэзээ нэг цагт хаях төлөвлөгөөтэй.
④ Одоогийн байдлаар тамхиа хаях санаа огт байхгүй.

Сэтгэцийн эрүүл мэндийн байдал
1. Та өдөрт дунджаар хэдэн цаг унтдаг вэ? ____________ цаг

2. Сүүлийн үед стрессийг хэр зэрэг мэдэрч байна вэ?
① Маш их ② Их ③ Жаахан ④ Бараг үгүй

3. Өнгөрсөн 2 долоо хоногийн хугацаанд доорх шинж тэмдэгээс хэр зэрэг илэрч байна вэ?
(Хамаарах хэсгийг V гэж тэмдэглэнэ үү)

Үзүүлэлт	Огт үгүй	Олон хоног	Долоогоос олон хоног	Бараг өдөр бүр
1) Ажил хийх сонирхол хүсэл бараг байхгүй.				
2) Ганихарч цөхрөн яахаа мэдэхээ байх				
3) Унтахад хэцүү байх эсвэл байнга сэрэх, эсвэл маш их унтах				
4) Ядрах, хүчгүй сульдах				
5) Хоолонд дургүй болох эсвэл хэт их идэх				
6) Би өөрийгөө муу хүн гэж мэдрэх эсвэл өөрийгөө ялагдагч гэж мэдрэх, надаас болж би өөрөө, миний гэр бүлийнхэнд муу зүйл тохиолдож байна гэсэн мэдрэмж				
7) Сонин унших, TV үзэхдээ төвлөрч чадахгүй байна.				
8) Бусад хүмүүс мэдэхүйцээр биеийн хөдөлгөөн, үг удаашрах. Мөн эсрэгээрээ хэт сандарч тайвширч чадахгүй хэвийн үеэс илүү их холхих				
9) Би ингэж байхаар үхсэн нь дээр гэх бодол, эсвэл ямар нэг байдлаар өөрийгөө хорлох бодлууд				

Биеийн хөдөлгөөн

'**Эрч хүчтэй хөдөлгөөн**' гэдэг нь идэвхитэй хөдөлгөөн хийснээр их амьсгаадах, зүрх хүчтэй цохилох хөдөлгөөнийг,
'**Дунд зэргийн хөдөлгөөн**' нь дунд зэргийн хэмээр хөдөлгөөн хийж бага зэргийн амьсгаадах, зүрх бага зэрэг хүчтэй цохилохыг хэлнэ.

1. Багадаа 10-аас дээш минут тогтмол их амьсгаадах, зүрх хүчтэй цохилдог эрч хүчтэй хөдөлгөөн, спорт, дасгал, иогийн дасгал хийдэг үү?
※ Жишээ нь: Гүйх, дээс тоглох, ууланд авирах, сагс тоглох, усанд сэлэх, бадимантоноор хичээллэх гэх мэт

① Тийм → 1-1. Ерөнхийдөө долоо хоногт эрч хүчтэй хөдөлгөөнтэй спорт, дасгал, иогийн дасгалыг хэдэн хоног хийдэг вэ? Долоо хоногт _______хоног
→ 1-2. Өдөрт эрч хүчтэй хөдөлгөөнтэй спорт, дасгал, иогийн дасгалыг хэдэн цаг хийдэг вэ?
өдөрт _______цаг _______минут

② Үгүй

2. Багадаа 10-аас дээш минут тогтмол бага зэрэг амьсгаадах, зүрх хүчтэй цохилох дунд зэргийн хөдөлгөөнтэй спорт, дасгал, иого хөдөлгөөн хийдэг үү?
※ Жишээ нь: Хурдан алхах, хөнгөхөн гүйх (гүйлт), weight training (булчингийн хөдөлгөөн), гольф, бүжгийн спорт, пилатес гэх мэт

① Тийм
→ 2-1. Ерөнхийдөө долоо хоногт дунд зэргийн хурдтай спорт, дасгал, иогийн дасгалыг хэдэн хоног хийдэг вэ? Долоо хоногт _______хоног
→ 2-2. Өдөрт дунд зэргийн хөдөлгөөнтэй спорт, дасгал, иогийн дасгалыг хэдэн цаг хийдэг вэ?
өдөрт _______цаг _______минут

② Үгүй

нийгмийн дэмжлэг
(Хамаарах хэсгийг V гэж тэмдэглэнэ үү)

	Огт байхгүй	Ихэвчлэн байхгүй	Ихэвчлэн байхгүй	Ихэвчлэн байхгүй	Ихэвчлэн байхгүй
1. Ярилцах шаардлагатай үед таны үгийг сонсож өгдөг хүн байдаг уу?	1	2	3	4	5
2. Таньд нөхцөл байдлыг сайн ойлгуулж мэдээллийг хүргэж чаддаг хүн байдаг уу?	1	2	3	4	5
3. Таньд хэцүү үед зөвлөгөө өгөх хүн байдаг уу?	1	2	3	4	5
4. Та өөрийнхөө бүх зовлон асуудлыг хуваалцдаг хүн байдаг уу?	1	2	3	4	5
5. Таньд заавал зөвлөгөөг нь авахыг хүсдэг хүн байдаг уу?	1	2	3	4	5
6. Та хамгийн нууц, санаа зовоосон асуудлаа хүртэл хуваалцах хүн байдаг уу?	1	2	3	4	5
7. Та өөрийн хувийн асуудлаа шийдвэрлэхэд зөвлөгөөг нь авдаг хүн байдаг уу?	1	2	3	4	5
8. Таны асуудлыг ойлгож өгдөг хүн байдаг уу?	1	2	3	4	5
9. Та орон дээр хэвтэрт орох тохиолдолд таньд туслах хүн байдаг уу?	1	2	3	4	5
10. Таныг өвдөх үед эмчрүү дагуулж яваад үзүүлээд өгөх хүн байдаг уу?	1	2	3	4	5
11. Та хоол хийж чадахгүй тохиолдолд таны өмнөөс хоолыг тань хийгээд өгөх хүн байдаг уу?	1	2	3	4	5
12. Таныг өвдсөн үед ар гэрийн ажилд тань туслах хүн байдаг уу?	1	2	3	4	5
13. Таньд хайр халамж үзүүлдэг хүн байдаг уу?	1	2	3	4	5
14. Таныг хайрлаж, өөртөө хэрэгтэй гэж боддог хүн байдаг уу?	1	2	3	4	5
15. Таныг тэвэрч, дэм өгөх хүн байдаг уу?	1	2	3	4	5
16. Таньтай хамт цагийг хөгжилтэй өнгөрөөх хүн байдаг уу?	1	2	3	4	5
17. Таньтай чөлөөт цагаа хамт хөгжилтэй өнгөрөөх хүн байдаг уу?	1	2	3	4	5
18. Таньтай сонирхолтой зүйлийг хамтдаа хийх хүн байдаг уу?	1	2	3	4	5
19. Таныг санаа зовсон асуудлаа орхиход тусалж чаддаг хүн байдаг уу?	1	2	3	4	5

Оролцооныхоо талаар маш их баярлалаа.
