# Supplementary material for: Relationship between socio-demographics, body composition, emotional state, and social support on metabolic syndrome risk among adults in rural Mongolia
Source: PLoS One. 2021 Sep 27;16(9):e0254141. doi: 10.1371/journal.pone.0254141 (PMC8475977; doi:10.1371/journal.pone.0254141)
Supplement: S2 Appendix — (PDF) [file pone.0254141.s002.pdf]

몽골 대사증후군 관리 기초 설문지 (Korean Version)

ID			
성별	남 ( ), 여 ( )		
신체계측	신장(키)	cm	
	몸무게(체중)	kg	
	복부둘레	cm	
	혈압(blood pressure)	mmHg	
	맥박	beats/min	
지질검사<br>Cholestech LDX	Glucose 혈당		
	Total Cholesterol<br>총콜레스테롤		
	Triglyceride<br>중성지방		

Memo	Inbody QR

몽골 대사증후군 관리 기초 설문지

일반적 특성

1. 귀하의 연령은? ______세

2. 귀하의 성별은? ①남 ②여

3. 귀하의 직업은?
   ① Professionals(선생님, 의사, 간호사, 변호사 등)
   ② Technicians and associate professionals
   ③ Clerks
   ④ Service workers
   ⑤ Skilled agricultural and fishery workers
   ⑥ 없다
   ⑦ 기타 ( )

4. 가족의 월평균수입은? ________________MNT

5. 귀하의 학력은?
   ①무학
   ②초등학교 졸업(중퇴 포함)
   ③중학교 졸업(중퇴 포함)
   ④고등학교 졸업(중퇴 포함)
   ⑤대학(원) 졸업(중퇴, 수료 포함)

6. 결혼상태는?
   ①배우자 있음
   ②배우자 없음

이환조사

1. 다음과 같은 질병으로 진단을 받았거나, 현재 약물 치료 중이십니까?

질병명	뇌졸중 (중풍)	심장병 (심근경색/ 협심증)	호흡기질환 (천식/폐렴)	고혈압	당뇨병	간 질환
진단여부						
약물치료여부						

기타 질환:____________________________

2. 평소에 당신의 건강은 어떻다고 생각하십니까?
   ① 매우 좋음 ② 좋음 ③ 보통 ④ 나쁨 ⑤ 매우 나쁨

음주

1. 다음은 최근 1년 동안의 음주(술) 경험에 대한 질문입니다.
1-1. 술을 얼마나 자주 마십니까?
① 최근 1년간 전혀 마시지 않았다
② 한달에 1번 미만
③ 한달에 1번 정도
④ 한달에 2-4번
⑤ 일주일에 2-3번 정도
⑥ 일주일에 4번 이상

1-2. 한번에 술을 얼마나 마십니까?
※ 소주, 양주 구분 없이 각각의 술잔으로 계산합니다. 단 캔맥주 1개 (355cc)는 맥주 1.6잔과 같습니다.
① 1-2잔
② 3-4잔
③ 5-6잔
④ 7-9잔
⑤ 10잔 이상

흡연

1. 현재 담배를 피우십니까?
① 매일 피움 → 1-1. 하루 평균 흡연량은 몇 개비 입니까? ______개비
② 가끔피움
③ 과거에는 피웠으나 현재 피우지 않음
④ 전혀 피운적이 없음

2. 앞으로 1개월 안에 담배를 끊을 계획이 있습니까?
① 1개월 안에 금연할 계획이 있다
② 6개월 안에 금연할 계획이 있다
③ 6개월 이내는 아니지만 언젠가는 금연할 생각이 있다
④ 현재로서는 전혀 금연할 생각이 없다

정신건강

1. 하루에 평균 몇 시간 잠을 잡니까? __________ 시간

2. 최근에 스트레스를 얼마나 느끼나요?
① 매우 많이 ② 많이 ③ 조금 ④ 거의 없음

3. 지난 2주 동안, 아래 나열된 증상들에 얼마나 자주 시달렸습니까? (해당 부분에 V표 하세요)

항목	전혀 아니다	여러날 동안	일주일 이상	거의 매일
1) 일을 하는 것에 대한 흥미나 재미가 거의 없음				
2) 가라앉은 느낌, 우울감 혹은 절망감				
3) 잠들기 어렵거나 자꾸 깨어남, 혹은 너무 많이 잠				
4) 피곤함, 기력이 저하됨				
5) 식욕 저하 혹은 과식				
6) 내 자신이 나쁜 사람이라는 느낌 혹은 내 자신이 실패자라고 느끼거나, 나 때문에 나 자신이나 내 가족이 불행하게 되었다는 느낌				
7) 신문을 읽거나 TV를 볼 때 집중하기 어려움				
8) 남들이 알아챌 정도로 거동이나 말이 느림. 또는 반대로 너무 초조하고 안절부절 못해서 평소보다 많이 돌아다니고 서성거림				
9) 나는 차라리 죽는 것이 낫겠다는 생각 혹은 어떤 식으로든 스스로를 자해하는 생각들				

신체활동

‘고강도 활동’은 격렬한 신체 활동으로 숨이 많이 차거나 심장이 매우 빠르게 뛰는 활동을,
‘중강도 활동’은 중간 정도의 신체 활동으로 숨이 약간 차거나 심장이 약간 빠르게 뛰는 활동을 말합니다.

1. 평소 최소 10분 이상 계속 숨이 많이 차거나 심장이 매우 빠르게 뛰는 고강도의 스포츠, 운동 및 여가 활동을 하십니까? [예시 카드를 참고 하세요]

※ 예: 달리기, 줄넘기, 등산, 농구 시합, 수영, 배드민턴 등

① 예 → 1-1. 평소 일주일 동안, 고강도의 스포츠, 운동 및 여가 활동을 며칠 하십니까?
일주일에 _______ 일
→ 1-2. 평소 하루에 고강도의 스포츠, 운동 및 여가 활동을 몇 시간 하십니까?
하루에 _______시간 _______분

② 아니오

2. 평소 최소 10분 이상 계속 숨이 약간 차거나 심장이 약간 빠르게 뛰는 중강도의 스포츠, 운동 및 여가 활동을 하십니까? [예시 카드를 참고 하세요]

※ 예: 빠르게 걷기, 가볍게 뛰기(조깅), 웨이트 트레이닝(근력 운동), 골프, 댄스스포츠, 필라테스 등

① 예 → 2-1. 평소 일주일 동안, 중강도의 스포츠, 운동 및 여가 활동을 며칠 하십니까?
일주일에 _______일
→ 2-2. 평소 하루에 중강도의 스포츠, 운동 및 여가 활동을 몇 시간 하십니까?
하루에 _______시간 _______분

② 아니오

사회적 지지

1. 질문을 읽고 가족, 친구, 이웃 등 주위의 사람들로부터 도움을 받고 있다고 느끼는 정도에 귀하가 가장 가깝다고 생각하는 곳에 체크(√)하여 주시기 바랍니다.

문항	전혀 아니다 1	대개 아니다 2	보통 이다 3	대개 그렇다 4	매우 그렇다 5
1. 내가 사랑과 돌봄을 받고 있다고 느끼게 해준다.					
2. 내가 취한 행동의 옳고 그름을 공정하게 평가해 준다.					
3. 내가 필요하다고 하면 아무리 돈이라고 해도 마련해 준다.					
4. 내가 그들에게 필요한, 가치 있는 존재임을 인정해 준다.					
5. 내가 하고 있는 일에 자부심을 가질 수 있게 나의 일을 인정해준다.					
6. 함께 있으면 친밀감을 느끼게 준다.					
7. 내 문제를 기꺼이 들어준다.					
8. 배울 점이 많은 존경할 만한 사람들이다.					
9. 자신이 직접 도움을 줄 수 없을 때는 다른 사람을 보내서라도 나를 도와준다.					
10. 내가 마음 놓고 믿고 의지할 수 있는 사람들이다.					
11. 내가 잘했을 때는(좋은 결과를 얻었을 때)칭찬을 아끼지 않는다.					
12. 나를 인격적으로 존중해준다.					
13. 무슨 일이건 대가(보상)를 바라지 않고 최선을 다해 나를 도와준다.					
14. 내가 어려운 상황(위기)에 직면했을 때 현명하게 문제를 해결할 수 있는 방안을 제시해 준다.					
15. 모두 의논할 문제가 생길 때 마다 나를 위해 시간을 내주고 응해준다					
16. 항상 나의 일에 관심을 갖고 걱정해 준다.					
17. 내가 몰랐던 사실은 일깨워주고 확실하게 해준다.					
18. 내가 결단을 못 내리고 망설일 때 결단을 내리게끔 자극을 주고 용기를 준다.					
19. 내가 현실을 이해하고 사회생활에 잘 적응할 수 있게끔 건전한 충고를 해준다.					

설문조사에 응해 주셔서 감사합니다.
