# Supplementary material for: Relationship between socio-demographics, body composition, emotional state, and social support on metabolic syndrome risk among adults in rural Mongolia
Source: PLoS One. 2021 Sep 27;16(9):e0254141. doi: 10.1371/journal.pone.0254141 (PMC8475977; doi:10.1371/journal.pone.0254141)
Supplement: Attachment — Submitted filename: Response to Decision Box.doc [file pone.0254141.s004.doc]

**<Author’ Responses to the Reviewer’ Comments>**

| Reviewer | Journal Requirements | Author Response to Comment |
| --- | --- | --- |
| Academic  Editor | Please ensure that your manuscript meets PLOS ONE's style requirements, including those for file naming. | According to the editor's request, it was revised throughout the manuscript in compliance with PLOS ONE journal style standards. |
|  | We note that you have indicated that data from this study are available upon request. PLOS only allows data to be available upon request if there are legal or ethical restrictions on sharing data publicly. We will update your Data Availability statement on your behalf to reflect the information you provide. If there are no restrictions, please upload the minimal anonymized data set necessary to replicate your study findings as either Supporting Information files. | We agree with PLOS ONE journal's data disclosure policy. According to the editor's opinion, we have attached data materials to the Supporting Information files. |
|  | Please include additional information regarding the survey or questionnaire used in the study and ensure that you have provided sufficient details that others could replicate the analyses. For instance, if you developed a questionnaire as part of this study and it is not under a copyright more restrictive than CC-BY, please include a copy, in both the original language and English, as Supporting Information. Moreover, please include more details on how the questionnaire was pre-tested, and whether it was validated. | We have attached the questionnaires (Mongolian version and Korean version) to the Supporting Information file at the request of the editor. The process of organizing the Mongolian version of the questionnaire was re-described in detail in the 'Measurements' section (Page 5, Line 119-126).  “All questionnaires were translated and back-translated into Mongolian. The research team consulted a Mongolian linguist and two nursing professors, all of whom were bilingual. One Korean lives in Mongolia and works as a professor of Korean studies at the Mongolian National University, and two Mongolian nursing professors have lived in Korean for years. They determined whether there was a difference in meaning. And if there is any disagreement in the translation-reverse translation process, the agreement was reached through discussion. The Mongolian version of the questionnaire has been further revised and supplemented with comments from experts consisting of two nursing professors and one social welfare professor. According to the results of a pilot study of 10 Mongolian residents, the Mongolian questionnaire was finally completed after being revised into easy-to-understand terms.” |

| Reviewer | Reviewer Comments | Author Response to Comment | | | Changes made to article | Page, line number |
| --- | --- | --- | --- | --- | --- | --- |
| Reviewer 1 | Kim et. al explored relationships between socio-demographics, clinical parameters, emotional state, and social support level on risk of prevalence of metabolic syndrome in Mongolia. Data was collected from voluntary clinic visits. The paper overall is well written; however there is concern with the stated goals and some of the conclusions. It is this reviewers suggestion to stick to the findings without making assumptions about how to reduce MetS in this area. In addition, the novelty was not clear as most of these correlations are well established. | Thank you for your valuable opinion. We completely agree with you. Our research team focused on metabolic syndrome in Mongolian rural areas (soum in the aimag) among local residents with a special life culture that consists of small communities and combines nomadic life. In Mongolia’s health care system, medical facilities and medical staff are heavily concentrated in Ulaanbaatar, the capital of Mongolia. Therefore, this study was conducted as a preliminary research to plan practical strategies for improving the health level of rural residents in Mongolia. Most previous studies have been conducted in countries or regions with generally good access to medical facilities and medical staff, and studies in Mongolia have been mainly conducted in Ulaanbaatar. However, local residents who we actually met in Mongolia said they were new to body composition measuring equipment or said they had performed blood sugar checks or urine tests years ago despite having diabetes. Thus, by identifying the factors related to metabolic syndrome among local residents living in areas with insufficient medical infrastructure and low access to medical care, we considered the planning of a health management strategy with limited resources. This is the novelty of our study. In addition, since biomarker indicators and psychosocial indicators (especially social support, depression indicators, etc) are used together, we think they can contribute to the construction of a multi-faceted intervention program for the management of metabolic syndrome in the future. We will fully reflect the valuable opinions and suggestions of the reviewer when planning follow-up research.  We fully agree with the reviewer’s comment. In the title, abstract, and purpose of the study, all expressions that can infer the “effects of an intervention” have been revised.  The revised sections were marked in red. | | | Title: “Relationship between socio-demo  graphics, body composition, emotional state, and social support on metabolic syndrome risk among adults in rural Mongolia”  Discussion section:  “Despite these limitations, this study targeted adults living in rural Mongolian regions with insufficient medical infrastructure and low access to medical care. In addition, since biomarker and psychosocial indicators (especially those of social support, depression, etc.) are used together, the findings could contribute to constructing multi-faceted intervention programs for the management of MetS in the future.” | Title  Page 1 Line1  Manuscript  thoroughly  Abstract  Page 2  Line 30  Page 4  Line 92-93  Line 98  Page 20  Line 426-  Page 21  Line 430 |
| Reviewer | Reviewer Comments | | Author Response to Comment | Changes made to article | | Page, line number |
| Reviewer 1 | Major Comments:  Line 92 (Stated goal of the paper): The study is all correlations innature. Therefore, stating that the study investigated the effects of socio-demographics… on the risk of MetS prevalence can’t be the goal.This would imply there is an interventions. Several of the conclusions are along the same lines. Without an intervention, you cannot say that a metastudy on depression and MetS that depression increases the prevalence of MetS without intervention. (Page 18, paragraph 3) | | Agreed and revised.  We fully agree with the reviewer’s comment. In the title, abstract, and purpose of the study, all expressions that can infer the “effects of an intervention” have been revised. In addition, the analysis results of previous review studies on the relationship between depression and metabolic syndrome have been described more clearly and amended as follows:  The revised parts were marked in red. | Title: “Relationship between socio-demo  graphics, body composition, emotional state, and social support on metabolic syndrome risk among adults in rural Mongolia”  Discussion section:  “According to literature reviews [22, 53] that examined links between MetS and depression, most cross-sectional studies reported higher depression scores among subjects with MetS than those without the condition. It is consistent with the results of this study, which showed relatively higher depression scores in groups with greater risk for MetS. Therefore, it is imperative that the management of psychological concerns, such as stress and depression, are considered in interventions developed to support adults in rural areas.” | | Title  Page 1 Line1  Manuscript  thoroughly  Abstract  Page 2  Line 30  Page 4  Line 92-93  Line 98  Page 19  Line 377-383  References  revised  [22]  Page 24  Line 519-521 |

| Reviewer | Reviewer Comments | Author Response to Comment | Changes made to article | Page, line number |
| --- | --- | --- | --- | --- |
| Reviewer 1 | Conclusion paragraph: It is hard to say that outsiders should “…ensure that they exercise regularly to reduce body fat and increase skeletal muscle.” While these thing do improve pathophysiology related to obesity, this usually isn’t effective long term as people tend to go back to their old lifestyle at some point. | Agreed and revised.  Thank you for your valuable opinion.  We recognize that it is very important for members of the community to participate directly in order to manage the health level of local residents in the long term. Dahlgren & Whitehead’s “Rainbow model” of multi-layered influences of health also explains that factors of individual health behavior are not independent but are influenced by multi-layered influences (local community, socio-economic, cultural, and environmental conditions, etc.). In this sense, an individual’s health care ability is influenced by the community, and most of the factors affecting health are considered to be more effective when approached at the community level. We fully agree that there are limitations to improving and sustaining a healthy lifestyle among local residents with one-time/short-term projects, such as outsiders and the ODA program. In other words, it is necessary for local residents to experience the process of identifying and solving health problems on their own through “participation.” Through this, the health care capacity of local residents will increase, and it might become an independent activity with sustainability. According to the reviewer’s opinion, the content of the conclusion has been revised as follows:  The revised parts were marked in red. | Discussion section:  “The characteristics and infrastructure of local communities play an important mediating role in the health of residents. Thus, given that an individual’s ability to manage their health and lifestyle is influenced by the community, several factors affecting health can be managed more effectively when approached from the local level. In this context, short-term strategies could consider resident participatory programs that engage and support rural community members in developing their human resources (e.g., health mentors). Among important long-term strategies, it is necessary to strengthen the competence of community health care professionals through systematic education and training for bagh feldsher and community nurses. Moreover, the status of rural primary health care services, such as family health centers, soum health centers, or intersoum hospitals, should be reviewed. In addition, it is necessary to establish a mid- to long-term health promotion policy by linking local networks with private resources, such as international volunteer organizations. As private projects, such as the official development assistant (ODA), often involve one-time visits or short-term programs, long-term and continuous intervention strategies must aim to support local residents' autonomy in health management. It is posited that such intervention strategies, consistently implemented over time, will be effective in reducing detrimental health and lifestyle practices.” | Page 18  Line 338-351. |
| Reviewer | Reviewer Comments | Author Response to Comment | Changes made to article | Page, line number |
| Reviewer 1 | Minor Comments  In the first paragraph of the results, the significant correlations between metabolic risk score and other variablesare stated, with a blanket statement for directionality (i.e. “variables tended  to be significantly higher.”) Authors should be definitive here. Does the voluntary nature of the study bias the data given that the study subjects had to get to the clinic? It seems less mobile people might be excluded since they might be less likely to travel. | Agreed and revised.  We re-described it specifically to improve the ambiguous representation of the research results.  Methods to minimize study bias that may occur due to voluntary participation are described in detail in the limitations of the study as follows:  The revised sections were marked in red. | Results section:  “Among participants with a high-risk score for MetS, higher values were parallelly observed in their demographic and clinical characteristics.”  Discussion section:  “First, as the convenient sampling method used in the study permitted participation of residents from specific rural areas, a limitation arises in generalizing and interpreting the research results for the total rural population of Mongolia. Certain measures were taken to encourage voluntary participation and address any study-related biases that participants may have. To minimize study bias that may occur due to voluntary participation, various methods were adopted. Local residents of the soum area (consisting of approximately 450 adults) were encouraged to participate through the cooperation of government officials who informed the residents about the study. In addition, participant convenience and comfort was considered and the main auditorium of the Office of Education, which has the best accessibility for local residents and is a largely familiar setting to them, was used for the purposes of data collection. Furthermore, following the advice of local Mongolians, data collection was conducted in July, which is regarded the best time for activities with minimal disturbances caused by extreme seasonal conditions, that also leads to movements characteristic of the local nomadic life. As the research team plans to conduct experimental interventions for the prevention and management of MetS for residents in this soum community in the future, efforts were made to collect basic data of as many residents as possible.” | Page 13  Line 283-289  Page 20  Line 405-418 |
