# Supplementary material for: Relationship between socio-demographics, body composition, emotional state, and social support on metabolic syndrome risk among adults in rural Mongolia
Source: PLoS One. 2021 Sep 27;16(9):e0254141. doi: 10.1371/journal.pone.0254141 (PMC8475977; doi:10.1371/journal.pone.0254141)
Supplement: Attachment — Submitted filename: Response to Decision Box.doc [file pone.0254141.s005.doc]

**<Author’ Responses to the Editor/Reviewer’ Comments>**

| Reviewer | Reviewer Comments | Author Response to Comment | Changes made to article | Page, line number |
| --- | --- | --- | --- | --- |
| Academic  Editor | Please review your reference list to ensure that it is complete and correct. If you have cited papers that have been retracted, please include the rationale for doing so in the manuscript text, or remove these references and replace them with relevant current references. Any changes to the reference list should be mentioned in the rebuttal letter that accompanies your revised manuscript. If you need to cite a retracted article, indicate the article’s retracted status in the References list and also include a citation and full reference for the retraction notice. The authors have addressed my comments mostly and improved the manuscript; however the conclusions in the abstract need to be about the data, not what the authors feel needs to happen to improve metabolic syndrome. Once this is addressed, I have no further comments and will recommend for publication. | Thank you for your careful review.  We found that the one retracted article (Reference list: 25. Kaur JA. Comprehensive review on metabolic syndrome. Cardiol Res Pract. 2014;1-21. doi: 10.1155/2014/943162.)  was included in a reference list. According to the editor's request, the retracted article removed and replaced with a new, relevant reference (Reference list: 25. Huang PL. A comprehensive definition for metabolic syndrome. Dis Model Mech. 2009;2(5-6):231-7. doi:10.1242/dmm.001180.)  The revised reference list was marked in red. | References section:  “ 25. Huang PL. A comprehensive definition for metabolic syndrome. Dis Model Mech. 2009;2(5-6):231-7. doi:10.1242/dmm.001180.” | Page 24  Line 527-528 |

| Reviewer | Reviewer Comments | Author Response to Comment | Changes made to article | Page, line number |
| --- | --- | --- | --- | --- |
| Reviewer 1 | The authors have addressed my comments mostly and improved the manuscript; however the conclusions in the abstract need to be about the data, not what the authors feel needs to happen to improve metabolic syndrome. Once this is addressed, I have no further comments and will recommend for publication. The authors have addressed my comments mostly and improved the manuscript; however the conclusions in the abstract need to be about the data, not what the authors feel needs to happen to improve metabolic syndrome. Once this is addressed, I have no further comments and will recommend for publication. | Thank you for your valuable opinion. We fully agree with the reviewer’s comment.  Based on the reviewer’s opinions, the conclusion in the abstract has been revised to focus on the main finding of this study.  The revised sections were marked in red. | Abstract  Conclusions:  “Our study demonstrated that MetS risk levels among adults living in rural Mongolia with limited medical resources were strongly associated with demographic characteristics, body composition and emotional health condition, particularly depression.” | Abstract  Page 2  Line 46-48 |
